# Effects of PB‐TURSO on the transcriptional and metabolic landscape of sporadic ALS fibroblasts

**DOI:** 10.1002/acn3.51648

**Published:** 2022-09-09

**Authors:** Jasmine A. Fels, Jalia Dash, Kent Leslie, Giovanni Manfredi, Hibiki Kawamata

**Affiliations:** ^1^ Feil Family Brain and Mind Research Institute, Weill Cornell Medicine 407 East 61st Street New York New York 10065 USA; ^2^ Neuroscience Graduate Program Weill Cornell Graduate School of Medical Sciences 1300 York Ave New York New York 10065 USA; ^3^ Amylyx Pharmaceuticals 43 Thorndike Street Cambridge Massachusetts 02141 USA; ^4^ Present address: Division of Biology and Biological Engineering California Institute of Technology Pasadena CA 91106 USA

## Abstract

**Objective:**

ALS is a rapidly progressive, fatal disorder caused by motor neuron degeneration, for which there is a great unmet therapeutic need. AMX0035, a combination of sodium phenylbutyrate (PB) and taurursodiol (TUDCA, TURSO), has shown promising results in early ALS clinical trials, but its mechanisms of action remain to be elucidated. Therefore, our goal was to obtain an unbiased landscape of the molecular effects of AMX0035 in ALS patient‐derived cells.

**Methods:**

We investigated the transcriptomic and metabolomic profiles of primary skin fibroblasts from sporadic ALS patients and healthy controls (*n* = 12/group) treated with PB, TUDCA, or PB–TUDCA combination (Combo). Data were evaluated with multiple approaches including differential gene expression and metabolite abundance, Gene Ontology and metabolic pathway analysis, weighted gene co‐expression correlation analysis (WGCNA), and combined multiomics integrated analysis.

**Results:**

Combo changed many more genes and metabolites than either PB or TUDCA individually. Most changes were unique to Combo and affected the expression of genes involved in nucleocytoplasmic transport, unfolded protein response, mitochondrial function, RNA metabolism, and innate immunity. WGCNA showed significant correlations between ALS gene expression modules and clinical parameters that were abolished by Combo treatment.

**Interpretation:**

This study is the first to explore the molecular effects of Combo in ALS patient‐derived cells. It shows that Combo has a greater and distinct impact compared with the individual compounds and provides clues to drug targets and mechanisms of action, which may underlie the benefits of this investigational drug combination.

## Introduction

ALS is a progressive and fatal neurodegenerative disorder that affects approximately every 5 per 100,000 people in the United States.^1^ The disease involves degeneration of both upper and lower motor neurons, causing muscle weakness and atrophy, spasticity, dysphagia, and neurocognitive symptoms. Eventual paralysis and death due to respiratory insufficiency typically occur within 2–5 years of diagnosis.^2^ More than 30 genes have been identified as major risk factors for ALS, and 10%–15% of ALS cases are associated with a causative underlying mutation in one or more of these genes, termed familial ALS (fALS). However, the majority of cases are sporadic (sALS) and have no defined underlying gene mutation.^3^

Diverse pathophysiological mechanisms have been proposed to contribute to motor neuron degeneration in ALS, including gliosis/inflammation, neuronal hyperexcitability, oxidative stress, disruptions in proteostasis, endoplasmic reticulum (ER) stress, alterations in RNA processing, abnormal stress granule dynamics, impaired vesicular transport, mitochondrial dysfunction, impairment in DNA damage repair, and alterations of nuclear transport.^2^ The pathophysiology of ALS likely results from a combination of these mechanisms, which converge to cause motor neuron degeneration. The lack of a clear genetic cause for sALS precluding the development of animal models and the etiological heterogeneity has thus far hindered the development of successful therapies. The only FDA‐approved drugs for ALS are Edaravone, which is thought to decrease oxidative stress, and Riluzole, which reduces excitotoxicity.^3^ The clinical efficacy of these treatments is very modest, and there is an urgent need for new treatments with a greater ability to slow functional decline and extend survival.

Recently, the combination of taurursodiol (also known as tauroursodeoxycholic acid, TUDCA, TURSO) and sodium phenylbutyrate (PB) was investigated in a double‐blind, randomized, multi‐site, placebo‐controlled phase 2 clinical trial (CENTAUR). The trial hit its primary endpoint, the rate of change in the ALS Functional Rating Scale.^4^ Importantly, the long‐term analysis showed that the median survival was increased by 6.5 months in the original treatment group compared with the placebo group.^5^ Moreover, the survival increased by 18.8 months for the original treatment group that continued treatment in the open‐label extension relative to the original placebo group of patients who did not enter the extension.^6^

TUDCA is a hydrophilic secondary bile acid produced by the conjugation of taurine to ursodeoxycholic acid. TUDCA is produced in the liver but can also be synthesized in the brain.^7^ Bile acids can act as signaling molecules, activating diverse pathways such as Nf‐κΒ, MAPK, PI3K, ERK, PLCγ, PKA, and Akt.^7^, ^8^, ^9^ They can act as a chemical chaperone and alleviate ER stress due to misfolded proteins^10^ and can block apoptosis by enhancing inner mitochondrial membrane integrity, reducing reactive oxygen species (ROS) production, and increasing oxidative phosphorylation.^11^ Additionally, it modulates epigenetics by reducing the activity and/or expression of histone deacetylases (HDACs) and histone acetyltransferases (HATs).^7^ TUDCA has shown anti‐inflammatory effects in several animal and cell models of neurodegeneration.^8^, ^12^

PB is an aromatized fatty acid that is metabolized into phenylacetate through β‐oxidation, which can be then conjugated to glutamine to form phenylacetylglutamine, which acts as an ammonia sink. PB is approved by the FDA for the treatment of urea cycle disorders.^13^ PB is an HDAC inhibitor and modulates chromatin remodeling and transcription by increasing histone acetylation.^14^, ^15^ PB is a chemical chaperone and is protective in encephalopathies caused by protein instability.^16^ Evidence shows that PB can ameliorate ER stress and modulate the unfolded protein response (UPR).^17^ In the SOD1 *G93A* mouse model of fALS, PB improves survival, motor function, and histone acetylation and reduces motor neuron loss, gliosis, ubiquitin‐positive aggregates, and apoptosis.^18^

TUDCA and PB are highly multifunctional molecules, and the breadth of cellular processes targeted by these two drugs could be beneficial in ALS. Their partially overlapping mechanisms of action suggest that in combination they may lead to synergistic activity. While each molecule individually has been the subject of several studies, the molecular effects of the TUDCA‐PB combination have not been studied. In this work, we compared the effects of TUDCA and PB (Combo) to those of each individual drug in skin fibroblasts from sALS patients and healthy controls using unbiased metabolomics and transcriptomics.

sALS fibroblasts have been shown to manifest metabolic and transcriptomic alterations^19^, ^20^, ^21^, ^22^, ^23^ and display altered age‐related metabolic profiles and bioenergetic states compared with control fibroblasts.^24^, ^25^ Furthermore, sALS fibroblasts show pathological changes similar to those seen in disease‐relevant cell types, including increased susceptibility to DNA damage, TDP‐43 cytosolic mislocalization,^26^, ^27^ and oxidative phosphorylation impairment.^28^ Moreover, perivascular fibroblasts from sALS patients exhibit transcriptomic alterations.^29^ This evidence indicates that fibroblast can be a viable platform to study the molecular effects of TUDCA‐PB.

We found that Combo caused many more changes in metabolism and gene expression than each individual drug and the effects of Combo were largely distinct from those of each drug alone. Combo‐modified genes are involved in mitochondrial function, UPR, intracellular trafficking/nucleocytoplasmic transport, innate immune function, nucleic acid metabolism, and RNA processing. While some of the changes were shared between sALS and CTL, others were differentially affected in sALS and CTL, providing new insight into the mechanisms of action of TUDCA and PB combination.

## Materials and Methods

### Cell culture and drug treatment

Twelve primary de‐identified fibroblast lines from healthy donors and 12 sALS patient lines were used in this study. The 24 cell lines were randomized from a larger group utilized by us in earlier studies^19^, ^20^, ^21^, ^22^, ^23^ and were matched for age at biopsy. Demographic and clinical data from all subjects are shown in Table 1. Cells were cultured in Dulbecco's modified Eagle medium (DMEM) containing 5 mM glucose, 2 mM l‐glutamine, 1 mmol/L sodium pyruvate, 10% FBS, and 1% penicillin/streptomycin. For all experiments, lines were assessed in passages 8–10. Treatment was done with 10 μmol/L TUDCA and 100 μmol/L PB for 5 days, replacing the medium with drug‐containing media daily. Doses are within the range of observed plasma concentration of the drug in patients according to unpublished data generated by Amylyx Pharmaceuticals.

**Table 1:** Demographic and clinical characteristics of de‐identified fibroblast lines used in this study.

	ID	Age at biopsy	Sex	Disease duration at the time of skin biopsy (months)	ALSFRS‐R total at biopsy	Rate of ALSFRS‐R decline	FVC %
Control	1	55	F				
	2	59	M				
	3	69	M				
	4	54	M				
	5	65	F				
	6	61	F				
	7	79	M				
	8	53	F				
	9	58	M				
	10	65	M				
	11	62	F				
	12	51	M				
sALS	1	64	M	14	35	0.93	79
	2	77	M	8	30	2.25	83
	3	67	F	14	35	0.93	78
	4	64	M	24	38	0.42	55
	5	41	F	9	30	2	90
	6	54	F	10	22	2.6	39
	7	64	F	15	28	1.33	22
	8	60	F	10	41	0.7	115
	9	64	M	15	27	1.4	45
	10	64	F	8	36	1.5	86
	11	68	F	12	24	2	98
	12	65	M	11	28	1.82	47

Age at biopsy average 60.9 years for CTL and 62.6 years for sALS (*p* > 0.6). Rate of ALSFRS‐R decline % calculated as ([48 − ALSFRS at Skin BX]/disease duration [months] at skin BX).

ALSFRS‐R, ALS Functional Rating Score‐Revised; sALS, sporadic; FVC, forced vital capacity.

### Statistical analyses of RNA sequencing and metabolomics

RNA Sequencing and Metabolomics methods are described in detail in Appendix S1, Supplementary Methods.

The R package DESeq2 version 1.24.0^30^ was used for normalization and differential gene expression analysis, with a low counts filter of <96 and all other filtering parameters kept as defaults. A Wald test was used to determine statistical significance, with the cutoff being a false discovery rate of <5% after Benjamini‐Hochberg correction. Weighted gene co‐expression network analysis was done using the normalized gene expression data from DESEq2 as an input for the functions included in the WGCNA package available from CRAN.^31^ We optimized parameters to maintain scale‐free topology and to allow for direct comparisons between the two networks, Q‐Q scaling was performed such that the 95% quantiles of both matrices matched. For all networks, the module merging parameter was kept consistent at 80%. Pairwise Pearson's correlations were used to calculate associations between modules and disease traits. Pathway analysis for all gene expression data was performed with the gprofiler2^32^ and clusterProfiler packages,^33^ using the gene ontology (GO) Molecular Function (GO:MF), GO Biological Process (GO:BP), and Kyoto Encyclopedia of Genes and Genomes (KEGG) databases. The cutoff for significance was an FDR corrected *p* value of <0.05. Pathways shown in the figures were condensed using the simplify function from the clusterProfiler package^33^ to merge terms with more than 40% overlapping annotated genes.

Relative metabolite abundance data were normalized with a log transformation, and differential abundance and pathway analyses were done with the free online tool MetaboAnalyst 5.0.^34^ Metabolite significance was determined with one‐way ANOVA with post hoc *t* tests, with the cutoff being a raw *p* value of <0.05, and the pathway significance cutoff was an FDR corrected *p* value of <0.05. Multiomics analysis was done with the mixOmics package.^35^ Data visualization was done in R using the ggplot2, pheatmap, corrplot, and venndiagram packages available from CRAN, and in GraphPad Prism version 9.3 (GraphPad Software, Inc). Z scores were calculated from normalized counts for each gene using the standard formula (*x* − *μ*)/*σ*, where *x* is the sample value, *μ* is the population mean, and *σ* is the population standard deviation.

## Results

### Combo has greater and distinct effects on metabolism than either PB or TUDCA alone

**Figure 1:**
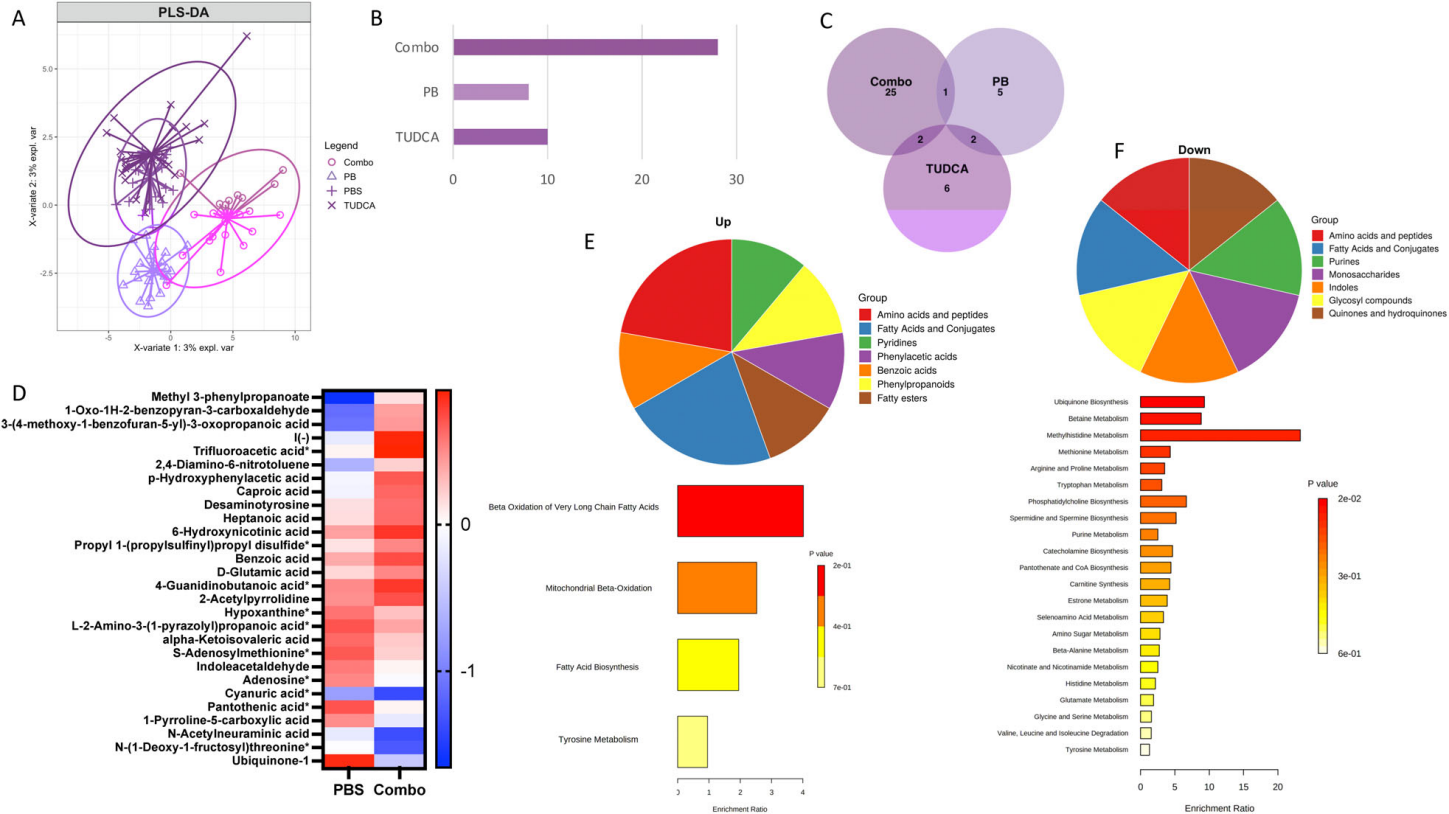
Combo changes more metabolites than either phenylbutyrate (PB) or taurursodiol (TUDCA) alone. (A) Partial least squares‐discriminant analysis of normalized abundance data for all metabolites. (B) Bar graph showing the number of significantly different metabolites (*p* value <0.05) for each treatment compared with vehicle. (C) Venn diagram of metabolites significantly changed by each treatment (*p* value <0.05). (D) Heatmap of *Z* scores of all metabolites significantly changed by Combo. *Indicates targeted metabolites. (E and F) Small Molecule Pathway Database category and pathway analysis of Combo‐upregulated (E) and Combo‐downregulated (F) metabolites.

For this study, we first examined the global effects of TUDCA, PB, and Combo on primary human skin fibroblasts, independent of the disease state (i.e., ALS or CTL). We performed unbiased metabolomics on cell lines (12 sALS and 12 CTL per treatment group) maintained in media containing physiological glucose levels (5 mmol/L) and identified 167 targeted and 631 untargeted polar metabolites (Table S1). Partial least squares‐discriminant analysis (PLS‐DA) of the 798 metabolites showed that samples treated with Combo were largely separable from the other treatments (Fig. 1A). Differential metabolite analysis identified 27 significantly different metabolites in Combo‐treated samples versus vehicle (PBS)‐treated samples, with only 10 and 8 significant metabolites with TUDCA and PB, respectively (Fig. 1B, Table S2). The majority (25/27) of significant metabolites in Combo were unique to this treatment (Fig. 1C). Therefore, Combo has distinct effects on metabolism that are not simply additive effects of PB and TUDCA.

Both targeted and untargeted metabolites changed by Combo are shown in Figure 1D. Combo‐upregulated metabolites included fatty acids, such as caproic and heptanoic acid, and methyl‐3‐propanoate. We note that identifiers assigned to the latter included PB derivatives, suggesting that its increase is due to drug metabolism. Downregulated metabolites included ubiquinone‐1, pantothenic acid, adenosine, s‐adenosylmethionine, and hypoxanthine. When Combo‐upregulated metabolites were grouped according to their Small Molecule Pathway Database (SMPDB) class, the majority were amino acids and fatty acids (Fig. 1E). Downregulated metabolites were split across several SMPDB classes, and approximately, half the significantly enriched pathways (12/22) were related to amino acid metabolism (Fig. 1F).

### Combo has greater and distinct effects on gene expression than either PB or TUDCA alone

**Figure 2:**
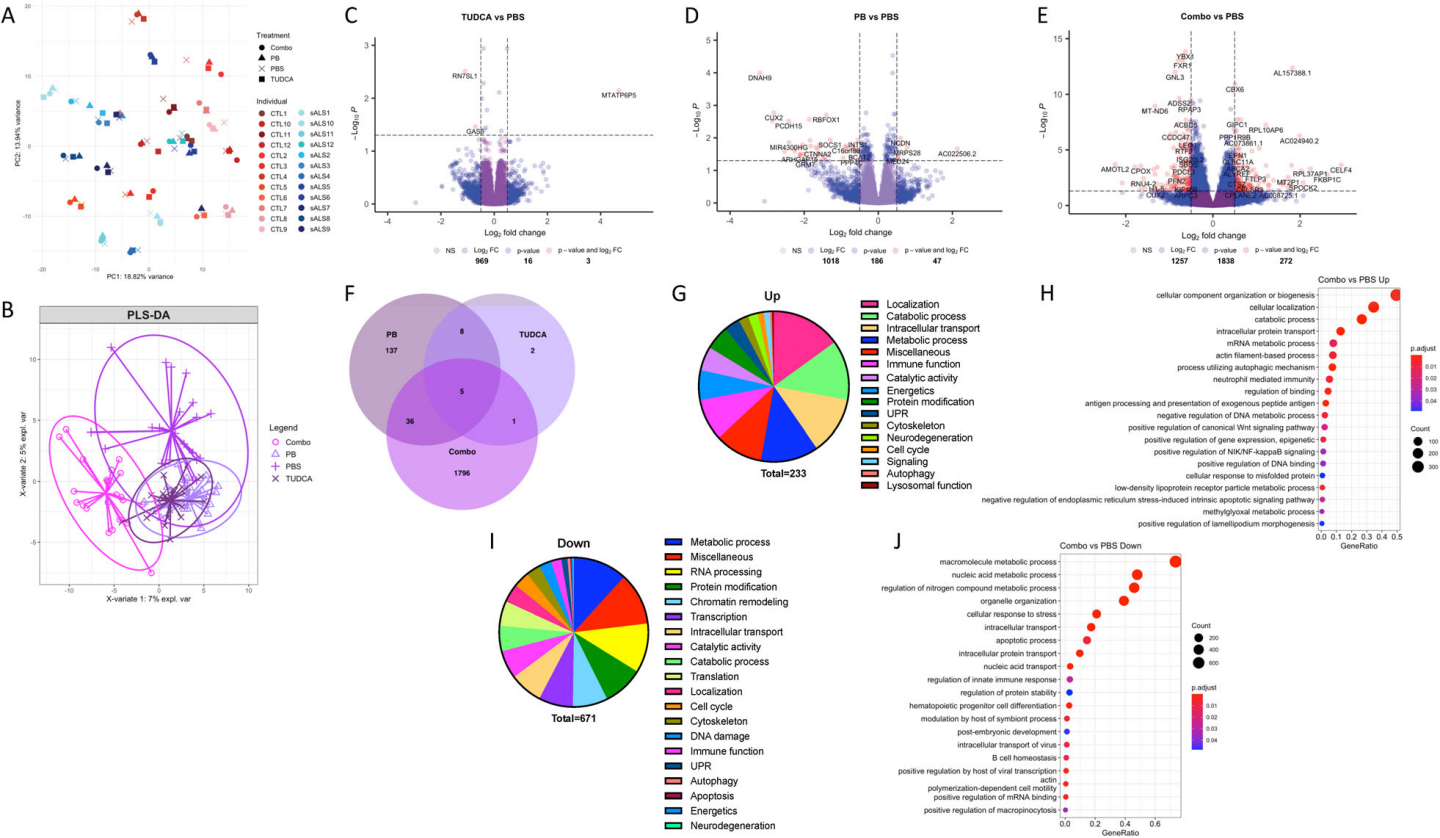
Combo changes more genes than either phenylbutyrate (PB) or taurursodiol (TUDCA) alone. (A) Principal component analysis of the top 500 genes with the highest variability. (B) Principal least squares‐discriminant analysis of the top 500 genes with the highest variability. (C–E) Volcano plots of differentially expressed genes (DEGs) of TUDCA (C), PB (D), and Combo (E) relative to the vehicle. (F) Venn diagram of DEGs in each treatment. (G) Category analysis of all significant gene ontology (GO) terms enriched in Combo upregulated DEGs. (H) Top 20 most significant GO:BP terms enriched in Combo‐upregulated DEGs, simplified with the clusterProfiler package. (I) Category analysis of all significant GO terms enriched in Combo downregulated DEGs. (J) Top 20 most significant GO:BP terms enriched in Combo downregulated DEGs.

Next, we performed unbiased 3′ RNA sequencing on total fibroblast RNA. We found that the major contributor to overall variability in gene expression was interline variability. Principal component analysis of the top 500 most variable genes showed that the factor driving clustering was the individual line identifier (Fig. 2A). However, PLS‐DA of the same genes showed that while PB and TUDCA samples overlapped with PBS‐treated samples, Combo samples could be separated (Fig. 2B). Therefore, similar to metabolomics, when samples were analyzed independent of disease state, Combo induced greater changes in overall gene expression than PB or TUDCA alone. Fewer differentially expressed genes (DEGs) (adj. *p* value <0.05) were found in the TUDCA versus PBS comparison (16 DEGs, Fig. 2C) and PB versus PBS (186 DEGs, Fig. 2D) compared with Combo versus PBS (1838 DEGs, Fig. 2E, Table S3). Similar to metabolites, the vast majority (1796/1838) of DEGs in the Combo versus PBS were unique (Fig. 2F).

GO analysis showed that among 233 pathways upregulated by Combo (Table S4), some of the most significant ones were related to intracellular transport, including nuclear transport (Fig. 2G and H). Several genes of the importin (*TNPO3*, *IPO5*, and *IPO7*) and nuclear pore complex (*TPR*, *NUP188*, *NUP153*, *NUP54*, and *POM121*) were changed by Combo (Fig. S1A). Other major categories upregulated by Combo included bioenergetic pathways, such as oxidative phosphorylation (Fig. 2G). Accordingly, genes encoding mitochondrial oxidative phosphorylation complexes were upregulated by Combo (Fig. S1B). Furthermore, another group of upregulated pathways was related to the UPR, including ER stress (Fig. 2G). Upregulated UPR genes included *WFS1* and *CREB3*, both of which promote cell survival under ER stress.^36^, ^37^ Of note, Combo is being studied for the treatment of Wolfram Syndrome with mutations in *WFS1*.^38^ *VCP*, which recruits ubiquitinated proteins to the proteasome and causes fALS when mutated,^39^ was also upregulated by Combo (Fig. S1C). Interestingly, we found upregulation of several genes associated with innate immune activation, in particular genes encoding subunits of the 20S and 26S immunoproteasome (Fig. S1D), which degrades antigens presented by MHC‐I, but may also participate in clearing misfolded proteins.^40^ Furthermore, STING1 was upregulated and cGAS downregulated by Combo (Fig. S1D). Overall, these findings show Combo upregulation of pathways associated with TUDCA and PB,^10^, ^11^, ^17^ such as mitochondrial function and ER stress, but also highlight novel pathways.

One of the largest categories of pathways downregulated by Combo of potential interest for ALS was RNA metabolism and processing^41^ (Fig. 2J and I and Table S4). Enriched terms encompassed processing of mRNA, rRNA, ncRNA, and miRNA and included polyadenylation, capping, and methylation activities. Other downregulated pathways were related to protein modifications, including acetylation, methylation, and polyubiquitination, as well as chromatin remodeling (Fig. 2I). Specific histone modification genes in downregulated pathways, encode for enzymes that perform H2B ubiquitination and H3 methylation at K36 and K4, marks associated with actively transcribed chromatin.^42^ Transcription‐associated pathways were also downregulated, and genes identified in these pathways included members of the mediator transcriptional coactivator complex, components of the regulatory machinery of RNA Pol II transcription. Taken together, GO analyses reveal a global downregulation of genes involved in transcription and RNA metabolism and processing.

To better understand the transcriptional regulation underlying Combo‐driven gene expression changes, we performed transcription factor (TF) binding site enrichment analysis on DEGs. The largest groups of TF‐binding sites enriched in both up‐ and downregulated genes belonged to Elk1, E2F1, and Elf1, while Sp1 and Egr1 were selectively enriched in upregulated genes, and YY1 and CREB1 in downregulated genes (Table S5). Elk1, E2F1, and Egr1 regulate cell cycle and apoptosis.^43^, ^44^, ^45^ Sp1 controls a broad range of cellular functions, including cell survival, immune responses, DNA damage responses, and chromatin remodeling.^46^ Elf1 regulates the immune response,^47^ and YY1 modulates DNA damage repair.^48^ The functions of these transcription factors align with the GO pathways identified in Combo‐regulated genes (Fig. 2), suggesting that these transcription factors are effectors of Combo treatment.

### Multiomics analysis identifies a set of metabolites and genes that discriminate Combo from vehicle treatment

**Figure 3:**
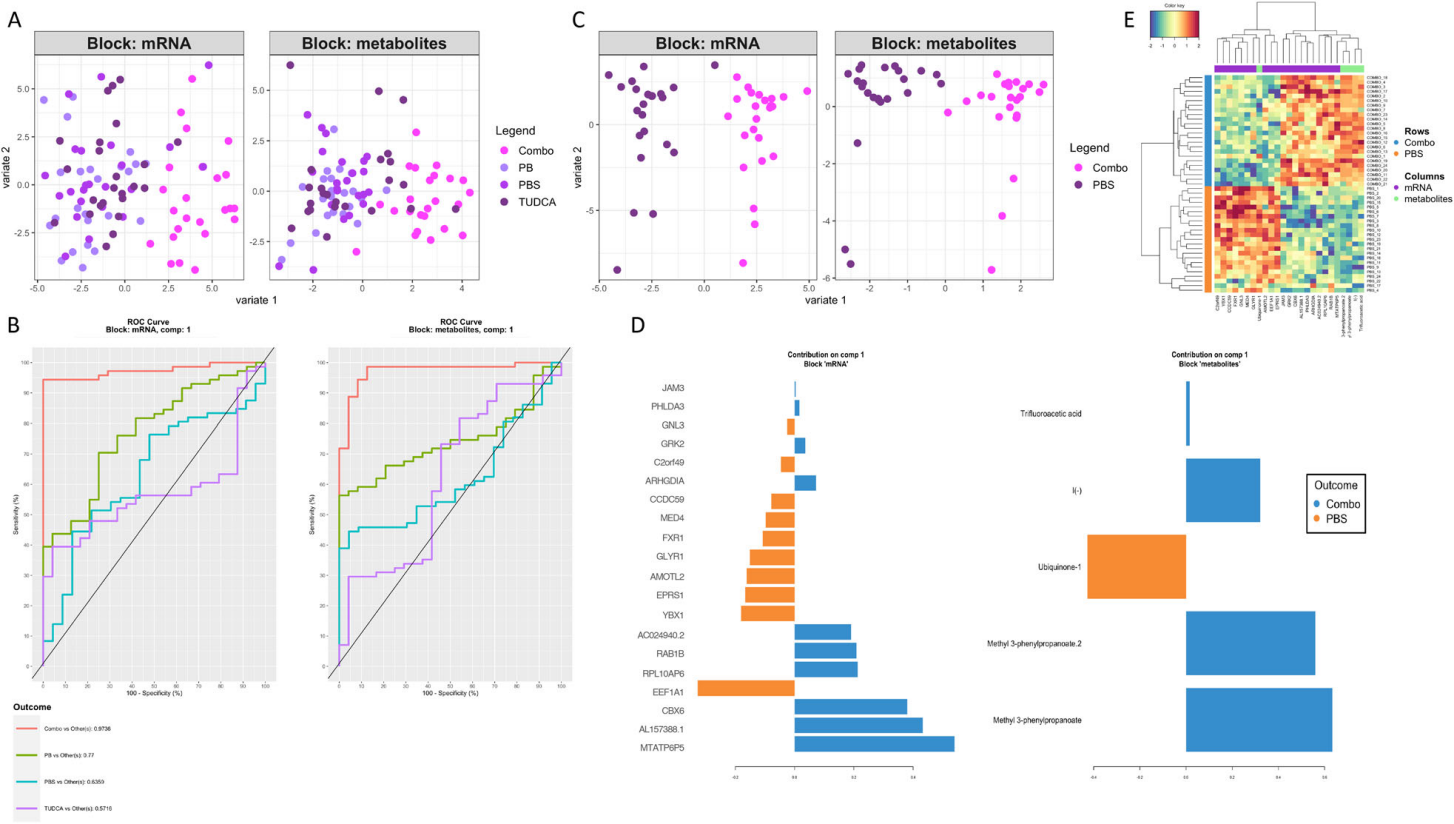
A set of 20 genes and 5 metabolites can discriminate Combo samples from the vehicle. (A) Principal least squares‐discriminant analysis of multiomics comparison normalized RNA and metabolites for all treatments, based on the top 20 most discriminatory genes and top 5 most discriminatory metabolites. (B) Receiver‐operating characteristic curves for the discrimination of each treatment from all other treatments based on the same 20 genes and 5 metabolites as in (A). (C) Principal least squares‐discriminant analysis of multiomics comparison normalized RNA and metabolites for Combo versus PBS based on the top 20 most discriminatory genes or top 5 most discriminatory metabolites. (D) Bar graphs of the top 20 genes and top 5 metabolites for Combo versus PBS. (E) Hierarchical clustering of samples based on the values of the 20 genes and 5 metabolites as in (C and D).

Next, we combined metabolomic and transcriptomic data sets for multiomics analysis to identify features able to distinguish Combo from the other treatments. We used the DIABLO (Data Integration Analysis for Biomarker discovery using Latent variable approaches for Omics studies) algorithm found by PLS‐DA that Combo‐treated cells (sALS and CTL combined) could be partially separated from all other treatments on Variate 1, which utilized the most discriminatory 5 metabolites and 20 genes (Fig. 3A). Receiver‐operating characteristic (ROC) curves showed that a model that uses these metabolites and genes discriminates Combo samples better than any other classification (Fig. 3B), confirming that Combo had more distinct effects than either drug individually. To further examine the genes and metabolites driving the classification of Combo from other groups, we performed the analysis using only Combo and PBS samples. PLS‐DA identified the most discriminatory 5 metabolites and 20 genes and showed that Combo could be fully separated from PBS on Variate 1 (Fig. 3C). Hierarchical clustering confirmed the full separation of Combo from PBS based on Variate 1 metabolites and genes (Fig. 3D). Several of the 20 genes encoded RNA‐binding proteins involved in RNA polymerase II transcription, which were decreased by Combo. The metabolites that most strongly discriminated Combo from PBS included methyl‐3‐propanoate/3‐phenylbutyric acid, ubiquinone‐1, and I hydroiodic acid. Taken together, this multiomics approach confirmed that Combo has unique effects on gene expression and metabolism, strongly driven by a subset of genes and metabolites identified with both methods of analysis.

### Combo has greater and distinct effects on gene expression in sALS compared with CTL fibroblasts

**Figure 4:**
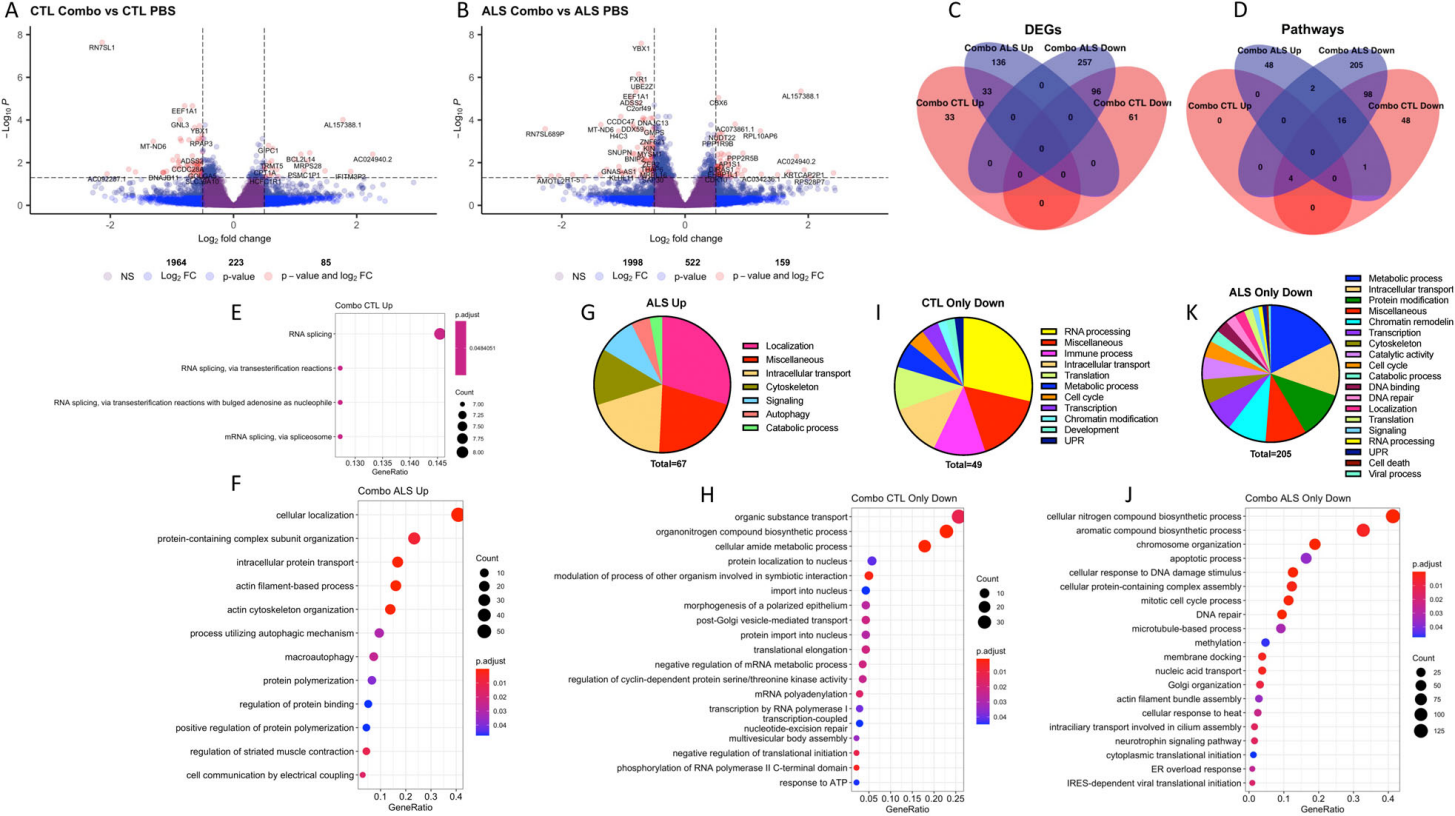
Combo has different transcriptional effects in sALS and CTL cells. (A and B) Volcano plots of differentially expressed genes (DEGs) in the Combo versus PBS comparison in CTL (A) and sALS (B) lines. (C and D) Venn diagram comparing DEGs (C) and gene ontology (GO) terms (D) changed by Combo in sALS and CTL lines. (E) Top 4 most significant GO:BP terms enriched in Combo‐upregulated DEGs in CTL. (F) Top 20 most significant GO:BP terms enriched in Combo‐upregulated DEGs in ALS. (G) Category analysis of all significant GO terms enriched in Combo‐upregulated DEGs in ALS. (H) Top 20 most significant GO:BP terms enriched in Combo downregulated DEGs in CTL. (I) Category analysis of all significant GO terms enriched in Combo downregulated DEGs in CTL. (J) Top 20 most significant GO:BP terms enriched in Combo downregulated DEGs in ALS. (K) Category analysis of all significant GO terms enriched in Combo downregulated DEGs in ALS.

Next, we tested if the effects of Combo were different in sALS and CTL fibroblasts. All metabolites significantly changed by Combo in sALS were also changed in CTL (Fig. S2). On the other hand, there were double the number of DEGs in sALS relative to CTL (522 vs. 223) (Fig. 4A and B, Tables S6 and S7). Half of the upregulated DEGs in the CTL Combo versus PBS comparison overlapped with those identified in sALS (33/66) and ~66% of the downregulated DEGs also overlapped (96/157). The majority of DEGs in the sALS Combo versus PBS comparison were unique to this group (Fig. 4C). The same pattern was evident for significantly enriched pathways (Fig. 4D). Only four pathways were enriched in upregulated DEGs in CTL Combo versus PBS, all related to RNA splicing (Fig. 4E, Table S8), while there were 67 enriched pathways in the sALS Combo versus PBS comparison (Table S9). Major categories of pathways upregulated by Combo in sALS included intracellular transport, cytoskeleton organization, and autophagy (Fig. 4F‐G). Within the intracellular transport category, many of the DEGs identified in the combined (sALS and CTL) analysis (Table S5) overlapped with those found specifically in sALS (Table S9). Uniquely upregulated genes in sALS included regulators of autophagosome formation and ER stress response.

The largest category of terms uniquely downregulated by Combo in CTL was RNA processing (Tables S8 and S9), including terms associated with RNA binding, splicing, and polyadenylation (Fig. 4H and I). Unique to sALS, the largest category of downregulated terms was metabolic processes, mainly related to nucleic acid metabolism (Fig. 4J and K). Furthermore, more genes and terms related to RNA polymerase II transcription were downregulated by Combo in sALS compared with CTL, including *SETX*, a helicase that modulates RNA polymerase II binding to chromatin, known to be associated with juvenile ALS.^49^

Taken together, GO results reveal that many of the transcriptional effects of Combo are unique to sALS. In particular, intracellular transport and RNA polymerase II transcription genes were modulated only in sALS.

### WGCNA identifies associations between modules and disease traits that are modified by Combo

Weighted gene co‐expression network analysis (WGCNA) can complement differential gene expression analysis methods. WGCNA considers highly similar groups of genes based on expression patterns across samples as sets of interconnected modules,^31^, ^50^ thereby creating a network of gene expression patterns that can be correlated to disease traits. This approach increases statistical power for identifying associations between traits and gene expression profiles. WGCNA has recently been applied to discover transcriptomic alterations in ALS spinal cord.^51^

**Figure 5:**
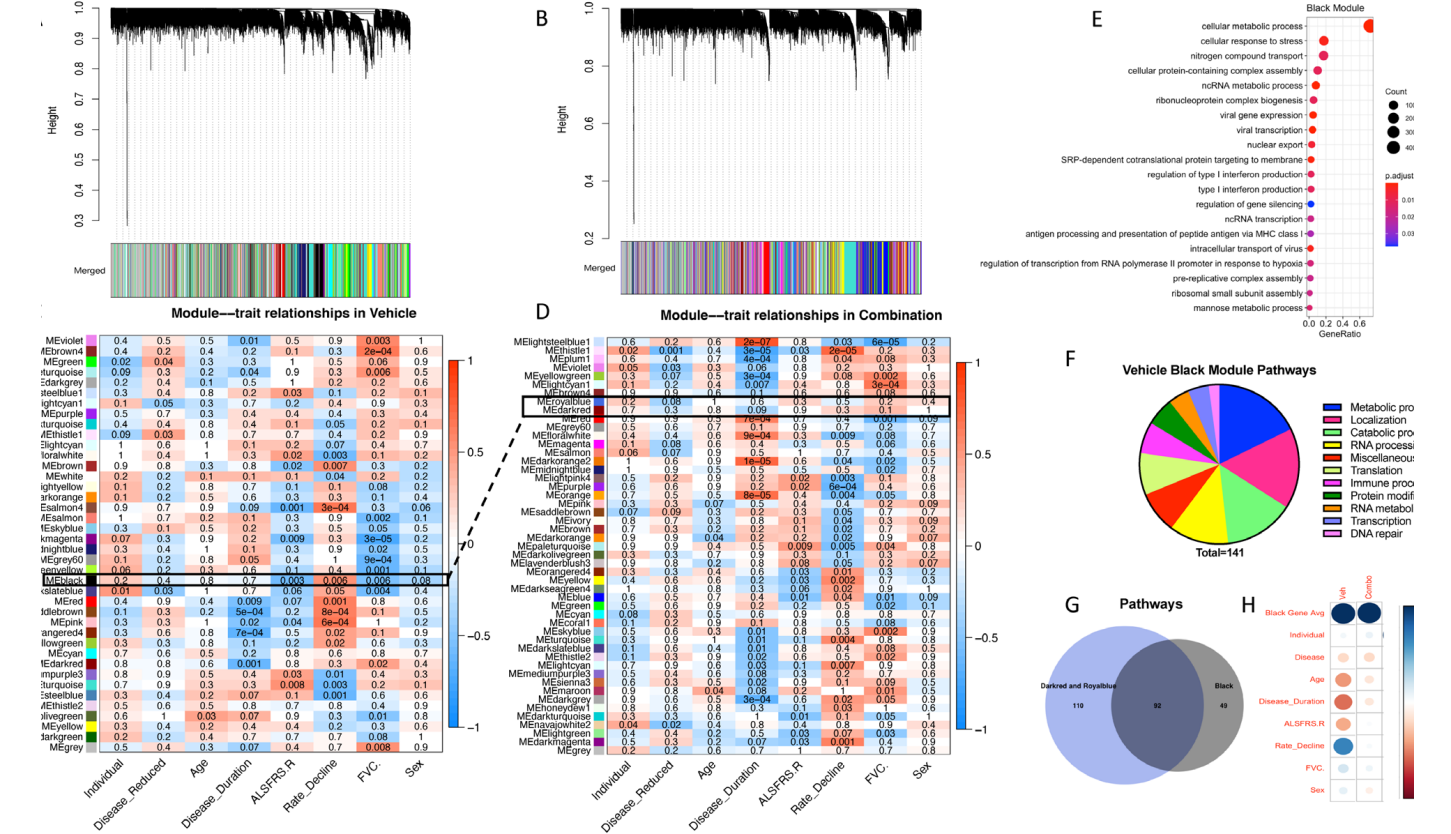
WGCNA identifies modules of genes associated with ALS clinical traits that are altered by Combo. (A and B) Dendrograms of hierarchical clustering of gene co‐expression modules for vehicle‐treated samples (A) and Combo samples (B). (C and D) Heatmaps showing correlations between module eigengene expression values and clinical traits (individual is a cell line identifier, disease reduced is a disease state, age is the age at biopsy, disease duration is the time between symptom onset and biopsy, ALSFRS.R is the score at the time of biopsy, rate_decline is the decrease in ALSFRS‐R/disease duration, FVC is %FVC at time of biopsy) for vehicle‐treated samples (C) and Combo samples (D). The individual cell line identifier was included as a negative control. The numbers in each box are *p* values, and box colors correspond to the correlation coefficient. (E) Top 20 most significant GO (gene ontology):BP terms enriched in the vehicle Black module. (F) Category analysis of all significant GO terms enriched in the vehicle Black module. (G) Venn diagram of overlap between significantly enriched GO pathways found in the vehicle Black module and the Combo modules (Darkred and Royalblue) paired with Black. (H) Correlations between the expression of the 758 Black module genes in vehicle and Combo samples and disease traits. Size correlates inversely to *p* value, and the color corresponds to Pearson's correlation coefficient.

We used normalized gene expression data from 16,492 genes that passed quality control filters as input to construct two co‐expression networks, one from all 24 Combo samples, and the other from all 24 vehicle (PBS)‐treated samples (Fig. 5A and B). After hierarchical clustering to identify groups of highly co‐expressed genes (modules), we found 49 modules in the Combo network and 40 modules in the vehicle network (Fig. 5C and D). We then correlated module gene expression with disease traits, including disease state (sALS vs. CTL), disease duration (time between symptom onset and skin biopsy), ALS Functional Rating Score‐Revised (ALSFRS‐R) score, rate of decline in ALSFRS‐R, and forced vital capacity (FVC)%. We also included age and sex as potential biologically relevant variables. In PBS samples, sex was not a major driver of variance in overall gene expression, while age significantly correlated with PC1 (Fig. S3). In Combo samples, sex and age did not correlate with any of the top three PCs (Fig. S3). Therefore, variance in gene expression associated with age that exists in vehicle‐treated cells was attenuated by Combo treatment.

We identified 22/40 modules that were significantly (*p*_adj_ <0.01) associated with one or more traits in the vehicle network and 23/49 modules in the Combo network (Fig. 5C and D). Of the 22 trait‐associated modules from the vehicle network, 15 had at least one significant GO enrichment (Table 2). Of the 23 from the Combo network, 18 had at least one significant enrichment (Table 3). To identify functional differences in disease trait‐associated modules in response to Combo, we compared the GO results from modules in each network. A larger portion of the vehicle trait‐associated modules, particularly Grey60, Salmon, and Violet, had enrichment for terms related to developmental processes and cell cycle (Fig. S4, Table S10). These pathways were not detected in Combo modules. Furthermore, several terms related to cell death caused by oxidative stress were identified in the Paleturquoise and Violet modules and were not found in Combo modules (Fig. S4, Table S10). Major enriched pathways emerging from Combo were immune activation (Turquoise and Darkturquoise modules) and translation, both cytoplasmic and mitochondrial (Plum1 and Blue modules, Fig. S4, Table S10). Within each disease trait, the majority of GO terms enriched in all associated modules were not shared between vehicle and Combo (Fig. S5). Therefore, the functional associations of sets of genes correlated with disease traits were modified by Combo.

**Table 2:** Modules in the vehicle network with a significant trait association and GO annotation.

Disease	Disease duration	ALSFRS‐R	Rate of decline	FVC
	Darkred	Black	Black	Black
	Violet	Salmon4	Salmon4	Violet
	Red	Turquoise	Turquoise	Salmon
	Saddle brown		Red	Grey60
			Saddle brown	Darkolivegreen
			Floral white	Gray
			Brown	Pale turquoise
			Pink	

ALSFRS‐R, ALS Functional Rating Score‐Revised; FVC, forced vital capacity; GO, gene ontology.

**Table 3:** Modules in the vehicle network with a significant trait association and GO annotation.

Disease	Disease duration	ALSFRS‐R	Rate of decline	FVC
Thistle1	Thistle1	Dark turquoise	Thistle1	Lightsteelblue1
	Lightsteelblue1	Pale turquoise	Darkmagenta	Yellowgreen
	Yellowgreen	Blue	Yellow	Lightcyan1
	Lightcyan1		Lightcyan	Red
	Red		Turquoise	Skyblue
	Skyblue		Orange	Maroon
	Turquoise		Pale turquoise	Blue
	Orange			
	Plum1			
	Darkorange2			
	Darkgray			

ALSFRS‐R, ALS Functional Rating Score‐Revised; FVC, forced vital capacity; GO, gene ontology.

To further investigate changes in the module–trait associations caused by Combo, we compared modules from each network based on their components with Fisher's exact test, considering two or more modules as a matched pair if the adjusted *p* value <0.05 (Fig. S6). Thirty‐one of 40 vehicle modules had a match in the Combo network. Notably, the Black module was associated with ALSFRS‐R, rate of decline, and FVC% in the vehicle network and was paired with the Darkred and Royalblue modules in the Combo network, but neither had significant associations with any disease traits. This pairing of modules from the two networks was the most interesting because it was the only one in which associations were lost due to the Combo treatment. GO analysis of the 758 genes that make up the Black module revealed strong enrichment of RNA processing and metabolism pathways across multiple classes of RNAs (Fig. 5E). Another major group of pathways enriched in genes from the vehicle Black module was intracellular localization/transport (Fig. 5F). Individual Black module genes that had strong correlations with disease traits included *SELENOO*, a mitochondrial redox‐sensitive selenoprotein,^52^ *LONP1*, a mitochondrial protease that degrades damaged proteins,^53^ *UGGT1*, which recognizes unfolded glycoproteins in the ER,^54^ *ORAI1*, an essential component of ER store‐operated calcium entry,^55^ and *TRAM2* and *TMEM147*, members of the ER translocon complex^56^ (Table S11). Finally, the most commonly identified transcription factors with binding sites enriched in genes from the Black module were Elk1, E2F1, Elf1, Sp1, and Erg1 (Table S12), the same transcription factors identified in the differential expression analysis. These results support and extend those obtained through differential expression analysis (Figs. 2 and 4), and point to RNA processing/metabolism, intracellular transport, and ER and mitochondrial homeostasis as pathways modified by Combo.

Last, as the Combo Darkred and Royalblue modules paired with the vehicle Black module (with 40/164 and 129/642 genes in common with Black, respectively, Fig. S6), but did not associate with disease traits, we tested whether the loss of the association was due to differences in the set of nonoverlapping genes from each module. We concluded that this was unlikely, as 92 of the 141 enriched pathways in the Black module were also found in the Darkred and Royalblue modules (Fig. 5G), suggesting that there were no major functional differences. Furthermore, as the overlap between the genes in the Black module and those in Darkred and Royalblue was not complete (589/758 Black module genes not found in either Darkred or Royalblue, Fig. S6), we took all 758 Black module genes and calculated correlations between their average expression in Combo samples and the disease traits. This approach confirmed that the strong correlation of this set of genes with disease traits in the vehicle was significantly reduced by Combo (Fig. 5H). Taken together, these results identify a set of highly co‐expressed genes, that strongly correlate with several measures of ALS disease severity, and are modified by Combo such that after treatment their expression no longer correlates with the disease traits.

## Discussion

ALS is a rapidly progressive, fatal disease, but despite a clear need for better treatment strategies, clinical trial outcomes in ALS have been largely unsuccessful. Factors potentially driving the failure of investigational treatments include delayed diagnosis due to heterogeneity of disease presentation, a lack of disease biomarkers, and the disconnect between disease etiology in animal models of fALS and human sALS cases. The search for new treatments continues despite these hurdles and preclinical and clinical evidence point to TUDCA and PB as potential therapies for ALS. TUDCA^57^ and PB^58^ were tested individually in Phase 2 clinical trials showing modest improvements. However, in the Phase 2 CENTAUR trial, the TURSO–PB combination (AMX0035) significantly slowed ALSFRS‐R decline^4^ and extended survival in the open‐label extension trials.^5^ TURSO–PB is currently under clinical investigation in the multicenter Phase 3 (PHOENIX) trial. While the clinical investigation of this drug combination is advancing, the molecular effects of the Combo in human cells have not yet been characterized.

In this study, we elucidate the transcriptomic and metabolic effects of the TUDCA–PB combination using unbiased approaches in primary skin fibroblasts from sALS patients and healthy controls. We compared the effects of Combo to each individual drug. Remarkably, Combo changed many more metabolites and genes than either TUDCA or PB alone, and most changes were unique to Combo. Among metabolites, S‐adenosylmethionine downregulation is of particular interest because of its involvement in glutathione biosynthesis and its impact on epigenetics due to its role as a methyl donor for histone and DNA methyltransferases.^59^ Numerous studies have linked changes in DNA methylation and histone posttranslational modifications to ALS,^60^ including in a large collection of induced pluripotent stem cell‐derived motor neurons.^61^ Furthermore, altered DNA methylation patterns were recently described in a large‐scale study of blood samples from ALS patients.^62^ Future studies investigating the epigenetic effects of TURSO–PB will expand upon these findings.

Overall, many genes and GO pathways that have been investigated in ALS were altered by Combo, for example, several nucleocytoplasmic transport (NCT) pathways. NCT is affected in ALS,^63^, ^64^ as several models of ALS showed abnormal nuclear membrane shape, nuclear pore complex protein loss or mislocalization, and dysregulated NCT dynamics.^65^ NCT pathways were both up‐ and downregulated by Combo, although the majority of significantly changed nucleoporin genes were downregulated. Interestingly, the transcriptional changes produced by Combo in NCT pathways were more pronounced in sALS than CTL lines, but the functional implications of these changes for ALS remain to be investigated.

Consistent with the known effects of TUDCA and PB against ER stress, we observed upregulation by Combo of genes promoting survival in ER stress conditions and downregulation of mediators of UPR signaling. Combo also increased the expression of several subunits of the mitochondrial respiratory chain and innate immune pathways involving cGAS/STING signaling. Moreover, subunits of the immunoproteasome were upregulated by Combo. Instead, several genes connected to RNA pol II transcription were strongly downregulated by Combo.

Other combination therapies, beyond TURAO‐PB, are currently under clinical investigation for ALS. Notably, the combination of nicotinamide riboside and pterostilbene (EH301), which are proposed to be activators of sirtuin 1, is presently in phase III clinical trial (NO‐ALS). We recently investigated the metabolic and transcriptional effects of EH301 in sALS and CTL fibroblast lines,^66^ so these effects can be compared with those of Combo. Most notable pathways uniquely regulated by EH301 and not by Combo included the cell cycle and protein translation, while the main pathways in common between the two treatments were related to mitochondrial function, ER stress response, DNA damage repair, and innate immunity. Pathways regulated by Combo but not by EH301 included NCT and other intracellular transport functions and RNA polymerase II‐dependent transcription. The pathways affected by both combinations could highlight shared therapeutic targets, while the distinct ones could be potential targets for personalized therapy.

WGCNA was used to correlate changes in gene expression patterns driven by Combo with ALS clinical parameters. In sALS fibroblasts, we found associations between innate immune pathways and ALSFRS‐R and FVC% in both the vehicle and Combo networks. The Black module was strongly correlated with disease duration, ALSFRS‐R, and FVC% in the vehicle network and significantly enriched for several immune‐related GO terms. Interestingly, WGCNA using data from postmortem ALS spinal cord samples showed a strong correlation between expression of mitochondrial oxidative phosphorylation and immune activation genes and disease.^51^ Importantly, in our fibroblast Combo network, the Darkred and Royalblue modules, which significantly matched as a pair with the Black module in the vehicle network, lost all associations with disease traits. The loss of these associations suggests that Combo affects the expression of inflammation‐related genes, which are strongly correlated with measures of disease severity. The similarities between the findings in fibroblast and spinal cord suggest that sALS fibroblasts share common transcriptomic alterations with affected tissues from ALS patients and could provide clues to understanding the therapeutic mechanisms of action of Combo in ALS.

Several questions remain to be answered in future work. This study was done in primary fibroblasts from sALS patients, as these cells are accessible and easily manipulated to study drug effects. The extent to which the findings from fibroblasts are recapitulated in motor neurons, the primary cell type affected in ALS will need to be investigated. Furthermore, we studied drug effects at a single time point. As treatment in patients will extend into months or years, the longitudinal effects of Combo should be a focus of future research. Finally, the methodology used in this study lends itself to an exploration of genes and metabolites that correlate with or predict a therapeutic response to Combo treatment, but this information is not yet available. As clinical trials progress and biological samples and patient response data become available, it will be possible to examine the relationship between gene expression and metabolism in patient‐derived samples and clinical effects.

In summary, this study is the first to report the transcriptomic and metabolomic effects of TURSO–PB combo in cells from healthy controls and sALS patients. We found that Combo alters the expression of genes involved in pathways linked to neurodegeneration, including mitochondrial function, UPR, nucleocytoplasmic transport, and immune activation. We propose that the modulation of these pathways could underlie the neuroprotective effects of TURSO–PB in ALS.

## Author Contributions

JAF performed all data analyses and interpretation and wrote the manuscript. JD performed experiments. KL designed the project. GM designed the project, interpreted results and wrote the manuscript. HK designed the project, interpreted results and wrote the manuscript.

## Conflict of Interest

This work was supported in part by a sponsored research agreement with Amylyx Pharmaceuticals. At the time when the work was performed, KL was employed by Amylyx.

## Supporting information


**Table S1** Full metabolite identifiers and masses.
**Table S2.** Significant differential metabolite abundance results for all treatments compared with PBS.
**Table S3.** Differential expression results for all treatments compared with PBS.
**Table S4.** Gene ontology enrichment results for Combo compared with PBS.
**Table S5.** Transcription factor enrichment results for Combo compared with PBS.
**Table S6.** Differential expression results for Combo compared with PBS in CTL lines.
**Table S7.** Differential expression results for Combo compared with PBS in sALS lines.
**Table S8.** Gene ontology enrichment results for Combo compared with PBS in CTL lines.
**Table S9.** Gene ontology enrichment results for Combo compared with PBS in sALS lines.
**Table S10.** Gene ontology enrichment results for trait‐associated modules in vehicle and Combo networks.
**Table S11.** Vehicle Black module genes and correlations with ALSFRS‐R.
**Table S12.** Transcription factor enrichment results for the vehicle Black module.

**Figure S1** Alterations by Combo treatment in the expression of genes in ALS‐relevant pathways. (A–D) Heatmaps of *Z* scores of differentially expressed genes changed by Combo in the nucleocytoplasmic transport (A), oxidative phosphorylation (B), unfolded protein response (C), and innate immune activation pathways (D).

**Figure S2** Combo does not have different metabolic effects in sALS and CTL cells. (A) Bar graph showing number of significantly different metabolites changed by Combo in ALS and CTL lines (*p* value <0.05). (B) Venn diagram of metabolites significantly changed by Combo in ALS and CTL.

**Figure S3** Correlation between gene expression principal components and clinical traits in vehicle‐ and Combo‐treated cells. (A and B) Plots showing correlation coefficients between clinical traits and the first 10 principal components derived from gene expression for the vehicle (A) and Combo (B) networks. Size corresponds inversely to *p* value, with X's denoting correlations that are not statistically significant (adjusted *p* value >0.05), and color corresponds to Pearson's correlation coefficient.

**Figure S4** Gene ontology (GO) pathways associated with clinical traits in vehicle and Combo networks. (A and B) Category analysis of all significant GO terms enriched in all significantly trait‐associated modules in the vehicle (A) and Combo (B) networks.

**Figure S5** Gene ontology terms associated with ALS disease traits change after Combo treatment. (A–D) Venn diagrams showing the overlap between all modules significantly associated with clinical traits from the vehicle and Combo networks, for disease duration (A), ALSFRS‐R (B), rate of decline (C), and forced vital capacity % (D).

**Figure S6** Matching of modules in the vehicle and Combo networks. The table shows the correspondence between modules identified in the vehicle (vertical axis) and Combo (horizontal axis) networks. *p* values are calculated using Fisher's exact test, color corresponds to −log_10_(*p* value). The numbers in each box are the number of overlapping genes found in a pair of modules. The black box identifies the vehicle Black module and its Combo paired modules Darkred and Royalblue.

**Appendix S1** Supplementary Materials.
