# Supplementary figures and images for: Effects of PB‐TURSO on the transcriptional and metabolic landscape of sporadic ALS fibroblasts
Source: Ann Clin Transl Neurol. 2022 Sep 9;9(10):1551–64. doi: 10.1002/acn3.51648 (PMC9539390; doi:10.1002/acn3.51648)

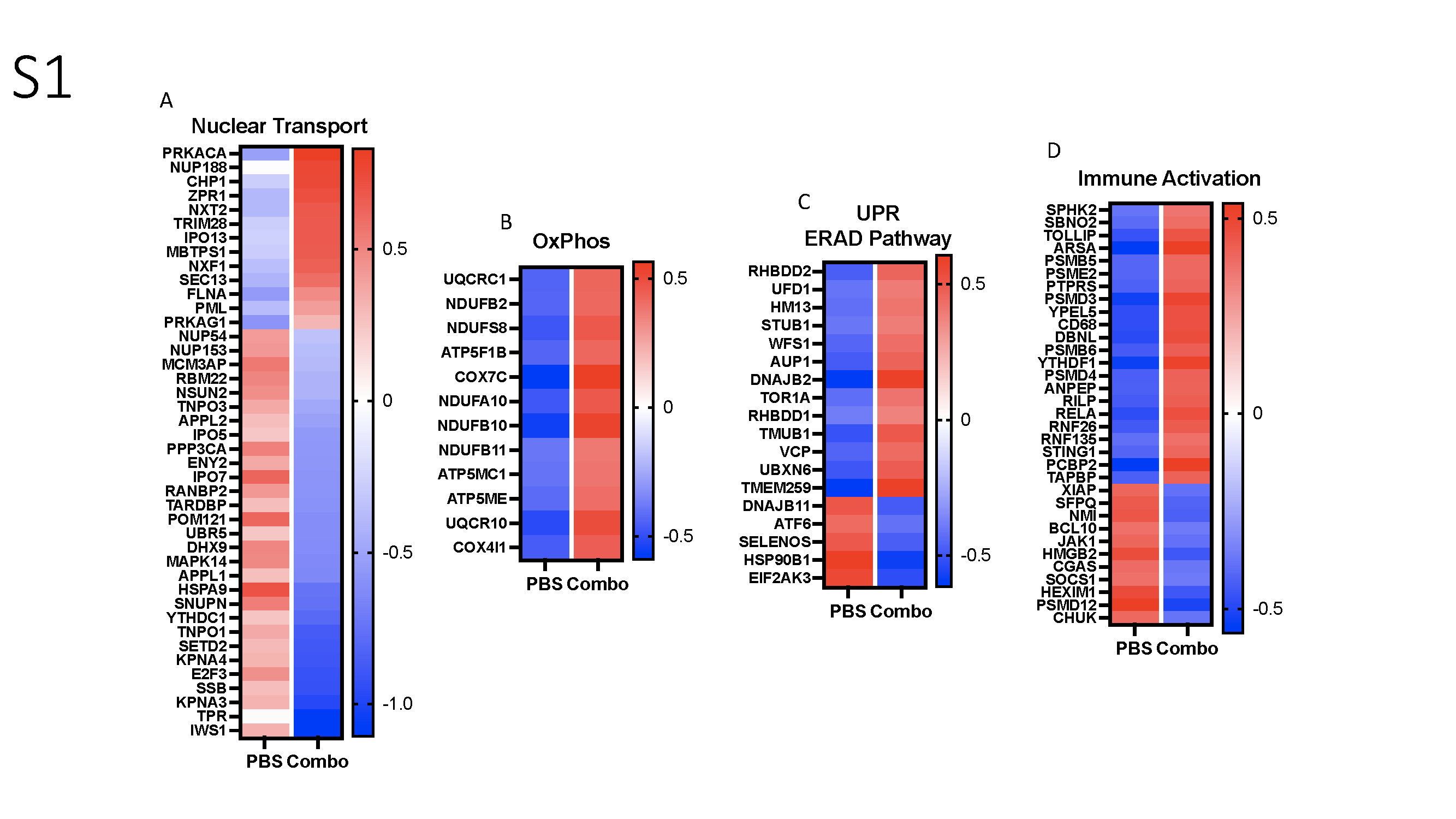

Supplement: Supplementary file 2 — Figure S1 Alterations by Combo treatment in the expression of genes in ALS‐relevant pathways. (A–D) Heatmaps of Z scores of differentially expressed genes changed by Combo in the nucleocytoplasmic transport (A), oxidative phosphorylation (B), unfolded protein response (C), and innate immune activation pathways (D). [file ACN3-9-1551-s008.tiff]

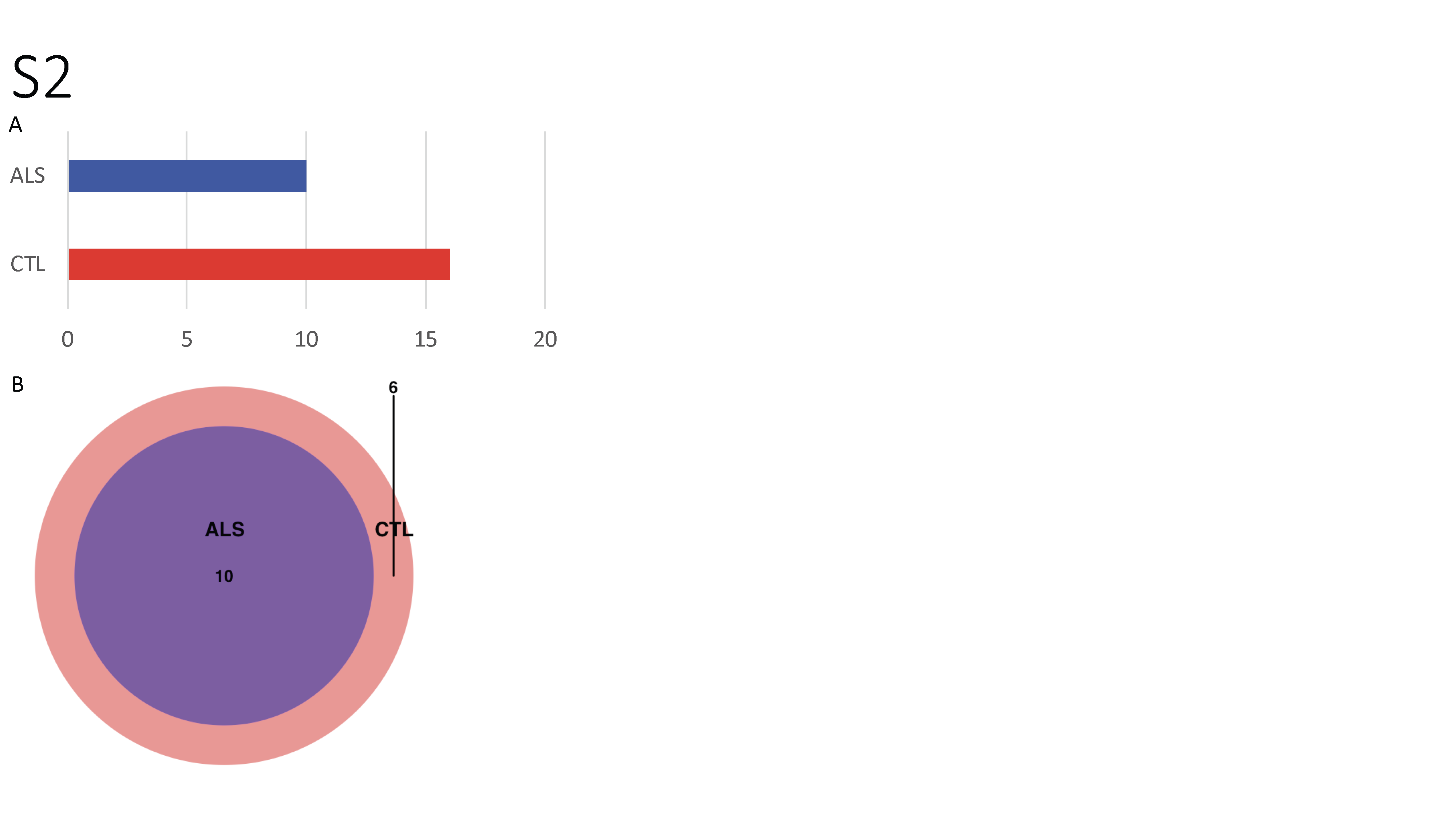

Supplement: Supplementary file 3 — Figure S2 Combo does not have different metabolic effects in sALS and CTL cells. (A) Bar graph showing number of significantly different metabolites changed by Combo in ALS and CTL lines (p value <0.05). (B) Venn diagram of metabolites significantly changed by Combo in ALS and CTL. [file ACN3-9-1551-s003.tiff]

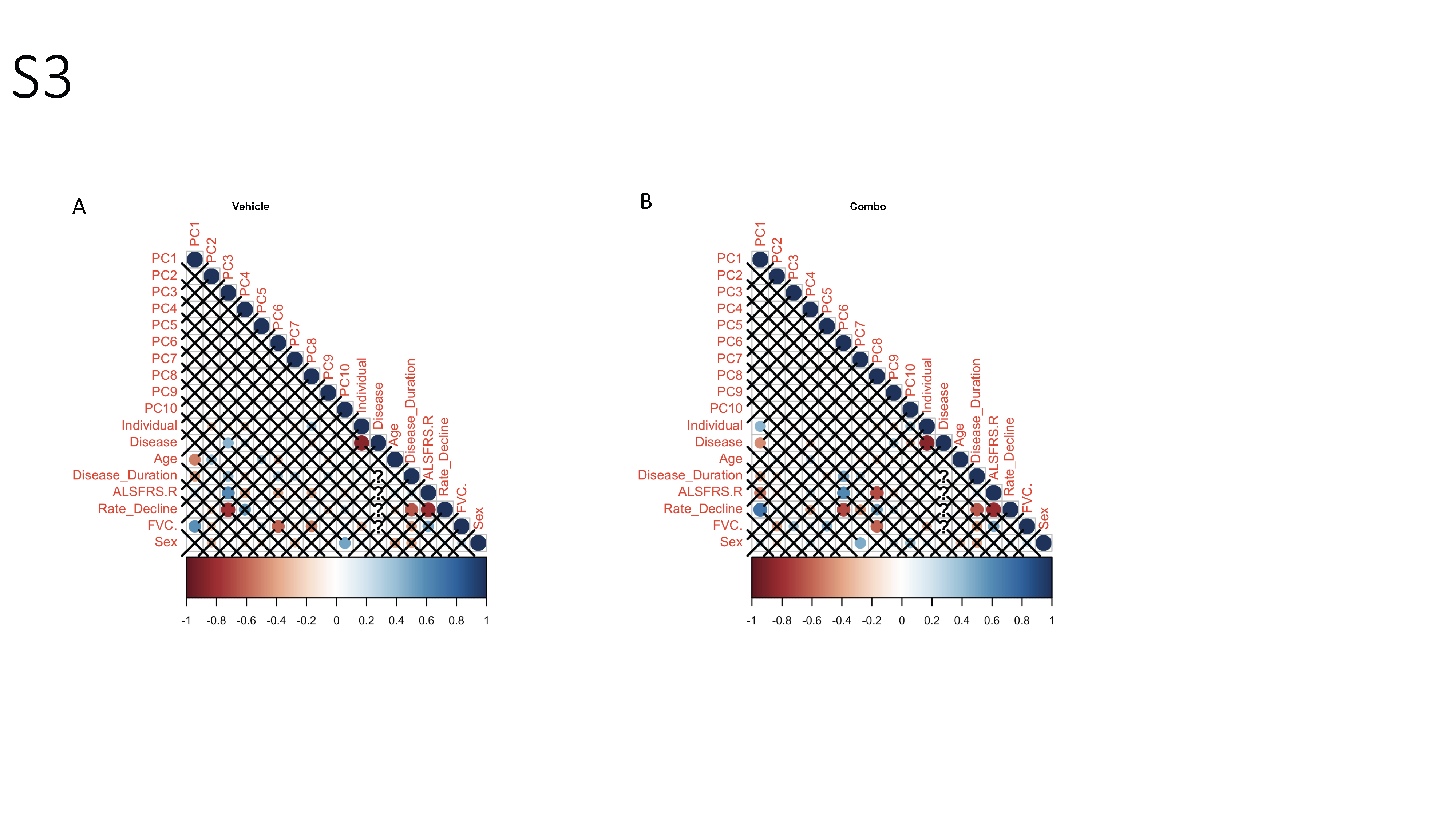

Supplement: Supplementary file 4 — Figure S3 Correlation between gene expression principal components and clinical traits in vehicle‐ and Combo‐treated cells. (A and B) Plots showing correlation coefficients between clinical traits and the first 10 principal components derived from gene expression for the vehicle (A) and Combo (B) networks. Size corresponds inversely to p value, with X's denoting correlations that are not statistically significant (adjusted p value >0.05), and color corresponds to Pearson's correlation coefficient. [file ACN3-9-1551-s002.tiff]

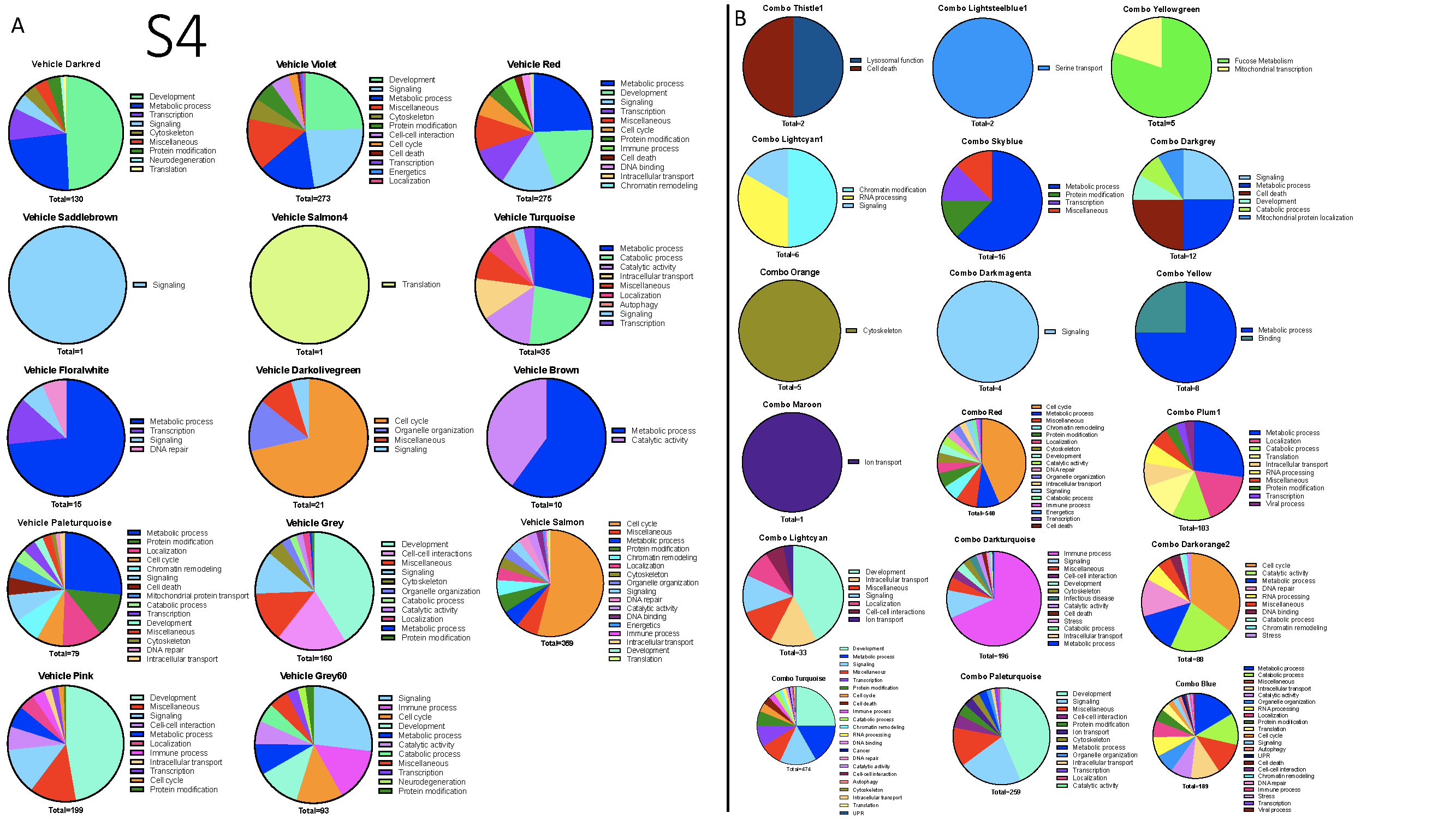

Supplement: Supplementary file 5 — Figure S4 Gene ontology (GO) pathways associated with clinical traits in vehicle and Combo networks. (A and B) Category analysis of all significant GO terms enriched in all significantly trait‐associated modules in the vehicle (A) and Combo (B) networks. [file ACN3-9-1551-s007.tiff]

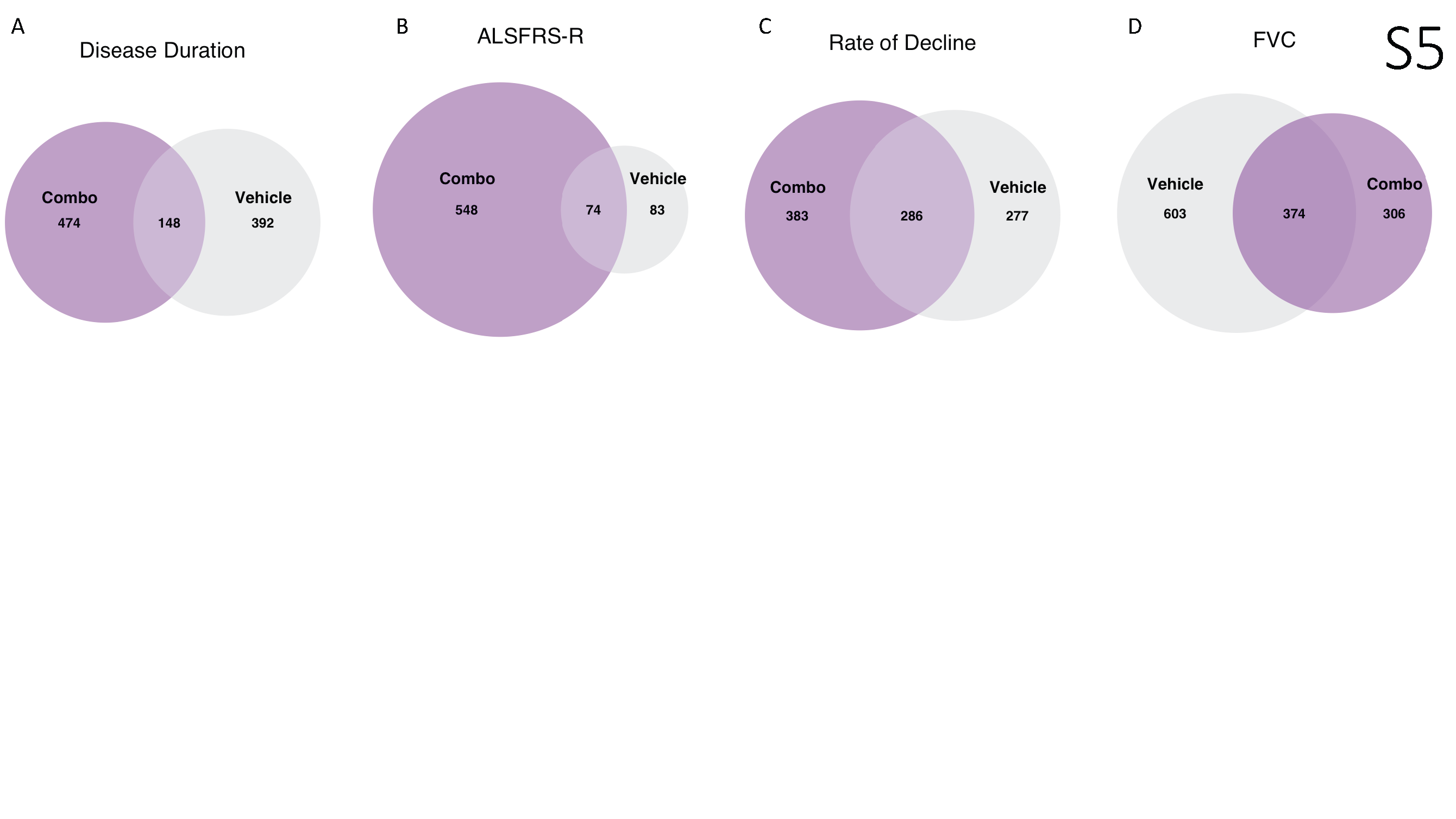

Supplement: Supplementary file 6 — Figure S5 Gene ontology terms associated with ALS disease traits change after Combo treatment. (A–D) Venn diagrams showing the overlap between all modules significantly associated with clinical traits from the vehicle and Combo networks, for disease duration (A), ALSFRS‐R (B), rate of decline (C), and forced vital capacity % (D). [file ACN3-9-1551-s005.tiff]

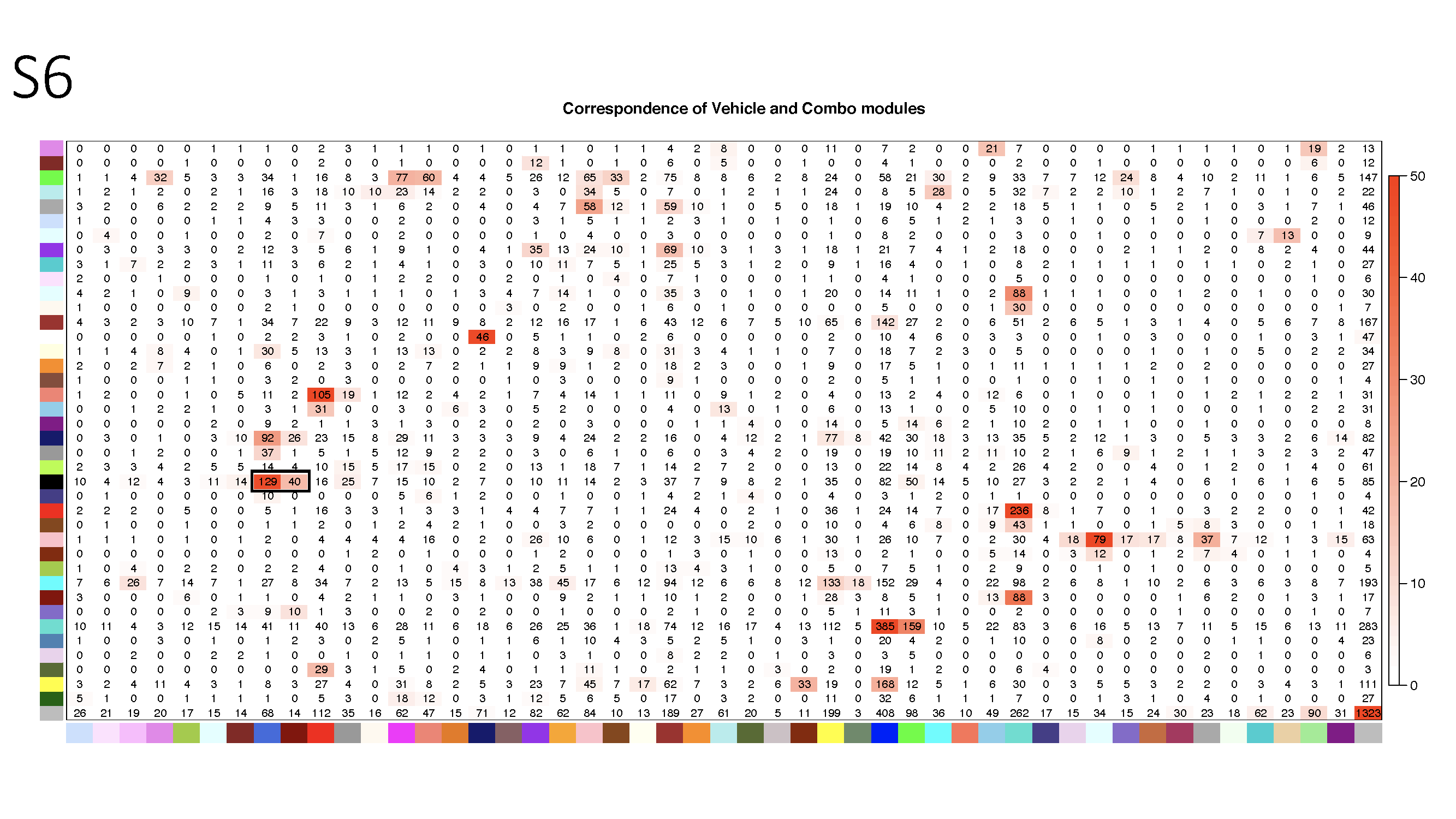

Supplement: Supplementary file 7 — Figure S6 Matching of modules in the vehicle and Combo networks. The table shows the correspondence between modules identified in the vehicle (vertical axis) and Combo (horizontal axis) networks. p values are calculated using Fisher's exact test, color corresponds to −log10(p value). The numbers in each box are the number of overlapping genes found in a pair of modules. The black box identifies the vehicle Black module and its Combo paired modules Darkred and Royalblue. [file ACN3-9-1551-s006.tiff]
