# Supplementary material for: Effects of PB‐TURSO on the transcriptional and metabolic landscape of sporadic ALS fibroblasts
Source: Ann Clin Transl Neurol. 2022 Sep 9;9(10):1551–64. doi: 10.1002/acn3.51648 (PMC9539390; doi:10.1002/acn3.51648)
Supplement: Supplementary file 8 — Appendix S1 Supplementary Materials. [file ACN3-9-1551-s001.docx]

Supplementary Methods

RNA Sequencing

Cells were harvested in Trizol (Thermo Fisher), homogenized through an 18g syringe, extracted with chloroform, and centrifuged at 12,000 x g for 15 minutes at 4ºC. Total RNA was isolated using the SV Total RNA Isolation System (Promega). The Genomics Facility at the Cornell Institute of Biotechnology used 500ng of RNA/sample for 3’RNA library preparation with the Lexogen QuantSeq 3’ mRNA-Seq Library Prep Kit FWD (Illumina), sequenced libraries on an Illumina NextSeq500 sequencer (single end 1x86bp), and de-multiplexed based upon six base i7 indices using Illumina bcl2fastq2 software (version 2.18). Illumina adapters were removed using Trimmomatic (version 0.36). Trimmed reads were aligned to the human genome assembly GRCh38.p13 using the STAR aligner version 2.7.0f, and transcriptome assembly was done using HTSeq-count version 0.6.1 ^30^.

Metabolomics

Metabolites were rapidly extracted in 80% ice-cold methanol. Extracted samples were vortexed twice, cleared by centrifugation at 14,000 x g for 20 minutes at 4ºC, and stored at -80ºC. The Weill Cornell Medicine Meyer Cancer Center Proteomics & Metabolomics Core Facility performed hydrophilic interaction liquid chromatography-mass spectrometry (LC-MS) for relative quantification of polar metabolite profiles for both targeted and untargeted metabolites. Metabolites were measured on a Q Exactive Orbitrap mass spectrometer (Thermo Scientific), coupled to a Vanquish UPLC system (Thermo Scientific) via an Ion Max ion source with a HESI II probe (Thermo Scientific). A Sequant ZIC-pHILIC column (2.1 mm i.d. × 150 mm, particle size of 5 µm, Millipore Sigma) was used for separation. The MS data was processed using XCalibur 4.1 (Thermo Scientific) to obtain the metabolite signal intensity for relative quantitation. Total protein, determined by BCA Assay, was used for normalization. Targeted identification was available for 167 metabolites based on an in-house library established using known chemical standards. The remaining 629 untargeted metabolites were assigned one or more identifiers based on their mass/charge ratio, and for clarity in figures are represented with the most commonly cited metabolite identifier, with full identifier lists available in Table S1. Identification required exact mass (within 5ppm) and standard retention times.
